# Sickness absence among privately employed white-collar workers during the COVID-19 pandemic; a prospective cohort study

**DOI:** 10.1186/s12889-025-21566-1

**Published:** 2025-02-10

**Authors:** Kristin Farrants, Mira Müller, Kristina Alexanderson

**Affiliations:** Division of Insurance Medicine, Department of Clinical Neuroscience, Karolinska Institutet, Stockholm, SE-171 77 Sweden

**Keywords:** Sick leave, COVID-19, White-collar workers, Private sector, Prospective cohort

## Abstract

**Background:**

The COVID-19 pandemic brought with it several changes, both regarding infection with COVID-19 itself as well as measures to combat the spread of infection, that might have affected sickness absence (SA) patterns. The aim was to explore whether incidence and length of SA changed between 2019, 2020, and 2021, as well as to determine factors associated with SA due to COVID-19 or COVID-like diagnoses among privately employed white-collar workers.

**Methods:**

A cohort of all privately employed white-collar workers in 2018 in Sweden (*n* = 1 347 778; 47% women) was followed prospectively during 2019, 2020, and 2021 using linked nationwide register data. We calculated numbers and proportions of people with incident SA (in SA spells > 14 days) due to COVID-19, COVID-like diagnoses (certain respiratory, infectious, and symptom-based diagnoses used in the beginning of the pandemic for suspected COVID-19), and all other SA, respectively, and mean number of SA days with somatic and mental diagnoses. Logistic regression was used to determine odds ratios (OR) and 95% confidence intervals for associations between prior diagnosis-specific SA, sociodemographic and work-related factors and incident SA due to COVID-19 or COVID-like diagnoses.

**Results:**

In 2020, 0.6% of the women and 0.3% of the men had incident SA due to COVID-19. For SA with COVID-like diagnoses, the corresponding proportions were 1.2% and 0.5%. The proportion of people with all other SA was stable across the years, at 8.1–8.4% for women and 3.7–3.9% for men. The mean number of SA days per person increased each year for somatic diagnoses but for mental diagnoses it decreased from 2019 to 2020 and increased to 2021 among women and men. Factors associated most strongly with incident SA due to COVID-19 or COVID-like diagnoses were low income (adjusted OR-range 1.36–5.67 compared to the highest income group) and prior SA due to COVID-like diagnoses (OR-range 4.67–5.31 compared to those with no such prior SA).

**Conclusions:**

A small proportion of privately-employed white-collar workers had incident SA spells > 14 days due to COVID-19 or COVID-like diagnoses. The factors associated with SA due to COVID-19 were similar to factors associated with SA due to other diagnoses in previous studies.

**Supplementary Information:**

The online version contains supplementary material available at 10.1186/s12889-025-21566-1.

## Introduction

The recent COVID-19 pandemic has exerted multifaceted impacts on various societal domains, encompassing the labour market, public health, and sickness absence (SA) dynamics [1–3]. In Sweden, there was an increase in SA throughout the initial half of 2020 in contrast to preceding years [4, 5], primarily attributable to COVID-like diagnoses, such as Viral infection of unspecified site, Acute bronchitis, Acute laryngitis and tracheitis, Acute upper respiratory infections of multiple and unspecified sites, Cough, Fever, or Pain in throat and chest, among others [4]. For instance, the incidence of SA spells exceeding 14 days in April 2020 doubled compared to in April 2019 [5]. Existent research predominantly focuses on either the broader labour market [3, 6, 7] or specific occupational sectors such as healthcare and social care [8, 9]. Conversely, investigations pertaining to SA among privately employed white-collar workers, even before the pandemic, are scarce in the literature [10–12] considering the size of this group and its importance on the labour market. Previous research has shown that there are differences in SA by sociodemographic factors, such as sex, age, birth country, educational level, family situation, and branch of industry, even among white-collar workers [10–13]. To what extent this was the case for SA due to COVID-19 or COVID-like diagnoses is still unknown.

During the pandemic, white-collar workers generally had more possiblitities to work from home [14], something that was recommended by the Swedish Public Health Agency [15]. Working from home not only meant that people were less exposed to being infected with COVID-19 or other infectious diseases, it also enabled individuals to work with (mild) symptoms of COVID-19, i.e., when they were required to be in quarantine according to the Swedish Public Health Agency’s recommendations [15]. Furthermore, it might also have enabled individuals with other disorders to remain in work without requiring SA, e.g., by enabling more breaks or not having to commute to and from work [16]. According to a report from the Swedish Social Insurance Agency, certain occupations within white-collar and office jobs had lower SA rates during the pandemic than before [5]. However, not all white-collar occupations can be done remotely, and there may have been other factors at play. To what extent privately employed white-collar workers followed the trends of the general working population, as well as which sociodemographic factors that were associated with SA due to COVID-19 and COVID-like diagnoses has so far not been studied.

**The aim** was to explore whether the incidence and length of SA changed between 2019, 2020, and 2021, as well as factors associated with SA due to COVID-19 or COVID-like diagnoses in 2020 among privately employed white-collar workers.

## Methods

This is a population-based prospective cohort study of SA among privately employed white-collar workers during the years 2019, 2020, and 2021, using different SA measures.

### Data and study population

The study population has been used for previous studies and is described in more detail in previous publications [10, 12]. We used microdata from three nationwide Swedish registers, linked at individual level: [1] Longitudinal Integration Database for Health Insurance and Labour Market Studies (LISA): to identify the source population and for information on age, sex, country of birth, type of living area, family situation, educational level, income, occupational code, sector, branch of industry, and migration; [2] MicroData for Analysis of the Social Insurance database (MiDAS): for information about SA spells > 14 days (dates, extent (full- or part-time), main SA-diagnosis) and disability pension (DP) (dates and extent); and [3] the Cause of Death register for information on year of death.

The study population consisted of all privately employed white-collar workers aged 18–67 years in 2018 with an income above 75% of the minimum yearly income to be qualified for SA benefits (8190 Swedish Krona, SEK) [10, 12]. Those who had full-time DP the whole year 2018 were excluded. Those who died (*n* = 2837) or emigrated (*n* = 7905) were excluded from the year following their death/emigration. In the analyses, we excluded individuals during the years when they did not have an income above 75% of the minimum level for SA benefits. The total study population included 1 347 778 individuals.

### Variables

Variables were chosen based on prior research showing an association with SA [10, 12, 17, 18]. We used information on the following variables in 2019: *Sex*: woman or man; *Age*: 19–24, 25–34, 35–44, 45–54, 55–64, or 65–67 years; *Country of birth*: Sweden, other Nordic country, other EU27 (the 27 countries that were part of the European Union in 2013 were used in order to maintain comparability with previous studies on the same population [10–13]), or Rest of the world (including those with missing information; *n* = 69, 0.006%); *Educational level*: elementary school (< 10 years) (including those with missing information; *n* = 4957, 0.4%), high school (10–12 years), or college/university (> 12 years); *Family situation*: married/cohabiting with children below the age of 18 at home, married cohabiting without children at home, single with children at home, or single without children at home (including those with missing information; *n* = 376, 0.03%); *Type of living area* [19]: large city (Stockholm, Gothenburg or Malmö), medium-sized town (> 90,000 inhabitants within 30 km of city centre), or small town/rural (< 90,000 inhabitants or rural); *Branch of industry* of the company where the individual was employed, based on the Swedish Standard for Industry Classification (SNI) categorised into the following six groups: manufacturing, services, transport, construction and installation, care and education, or commerce and hospitality; *Company size*: micro (1–9 employees), small (10–49 employees), medium (50–249 employees), large (≥ 250 employees); *Income from work*,* sickness absence*,* and parental leave* was categorised based on the price base amount (PBA, a yearly sum, set by the Swedish government to index amounts of money adjusted for inflation) into the following seven groups: 0.18-<4 PBA (8190 − 181 999 SEK), 4-<7.5 PBA (182 000-341 249 SEK), 7.5-<10 PBA (341 250–454 999 SEK), 10-<12.5 PBA (455 000-545 999 SEK), 12.5-<15 PBA (546 000-682 499 SEK), 15-<17.5 PBA (682 500–796 249 SEK), or ≥ 17.5 PBA (≥ 796 250 SEK).

We used the International Classification of Diseases, tenth revision (ICD-10) [20] to categorize the SA diagnoses into mental (ICD-10: F00-F99 and Z73) and somatic (all others, excluding Z00-Z99). Missing information on diagnoses were treated as a separate category. We further divided SA diagnoses into the following more detailed categories (See Table 1):

**Table 1:** Diagnosis categories used in the study and the ICD-codes included in each category

Diagnosis category	ICD-10 codes
Depression	F32-F34, F38
Anxiety	F40-F42
Exhaustion disorder/burn out	F43.8, Z73.0
Other stress-related diagnosis	F43.0, F43.1, F43.2, F43.9
Other mental diagnosis	F00-F99 except the above
Musculoskeletal diagnosis	M00-M99
Injury	S00-T98, V01-Y98
Cancer	C00-D48
Cardiovascular diagnosis	I00-I99
Pregnancy-related diagnosis (for women)	O00-O99, N96
COVID-19	U07, U09, U10
COVID-like diagnosis, according to the definition of the Social Insurance Agency of what they term COVID-related diagnoses [5]	J00, J02, J04, J06, J11, J12, J16, J18, J20, J21, J22, J44, J45, J46, J80, J96, J98, A08, A09, B09, B34, B97, B99, R00, R05, R06, R07, R20, R21, R23, R43, R50, R51, R53, R65
Other somatic diagnosis	all others, excluding Z00-Z99
Information on diagnosis is missing	information about the diagnosis was missing in the MiDAS register

### Public sickness absence insurance in Sweden

The Swedish public SA insurance system has been described previously [10, 12, 13]. In brief, all people living in Sweden with a work income can receive SA and DP benefits if their work capacity is reduced by morbidity. After a first qualifying day, during which no benefits are paid (self-employed can have more qualifying days), the employer provides sick pay for days 2–14, after which the Social Insurance Agency pays SA benefits. Unemployed persons get SA benefits from the Social Insurance Agency after the first qualifying day. A physician certificate is required after 7 days of self-certification, although during the COVID-19 pandemic this was temporarily changed to 21 days. We did not include SA spells < 15 days in the study, so as not to introduce bias regarding those who might have been unemployed. All residents in Sweden aged 19–64 years, whose work capacity is permanently or long-term reduced due to disease or injury, can be granted DP from the Social Insurance Agency. For many individuals with long-term SA (> 90 days) or DP, collectively bargained compensation is available from insurance companies, with one company managing insurances for privately employed white-collar workers, one company managing insurances for privately employed blue-collar workers as well as municipal and regional employees regardless of occupation, and one company managing insurances for state employees [21].

Both SA and DP can be granted for part- or full-time (25, 50, 75, or 100% of ordinary work hours). SA benefits cover 80% and DP benefits 64% of lost income, both up to a certain level.

### Statistical analyses

Descriptive analyses were conducted regarding numbers and proportions of people with at least one incident SA spell each year, as well as of mean number of annual net days with SA, in general and due to specific diagnoses. This latter measure was calculated first for all in the cohort, and secondly per person with SA each respective year, that is, two different types of denominators. For the calculation of net days, all prevalent SA spells were included, and part-time SA days were combined, e.g., two days of 50% SA or DP were combined to one net day. As we did not have information about the SA extent for the first 14 days, net days for the first 14 days of SA spells were calculated as being of the same extent as day 15 (e.g., if day 15 was at 50% absence, days 1–14 were counted as 7 net days).

We also conducted logistic regression to determine crude and adjusted odds ratios (OR) of having at least one incident SA spell due to COVID-19 or to COVID-like diagnosis during 2020, for socio-demographic, work-related, as well as prior SA-diagnosis factors (all measured in 2019). All variables were included in the adjusted analyses. Sensitivity analyses were run where we only included those who had SA spells with a diagnosis of COVID-19 in the outcome, i.e., those with only SA spells due to COVID-like diagnoses were not included.

We present the results for all people included, as well as stratified by sex.

## Results

In Table 2 information about the cohort’s sociodemographic characteristics, work-related factors, and SA diagnoses in 2019 is presented for all and stratified by sex. The cohort included slightly more men than women, with women comprising 46.98%. The most common age group for both women and men was 45–54 years, representing 26.89% of the women and 28.20% of the men. Slightly more than half had at least some university education. The majority were born in Sweden and lived in cities. Nearly half of the persons in the cohort worked for big companies (those with more than 250 employees), with the largest branch of industry being services. The predominant income group for women was 7.5-<10 PBA, while for men it was 10-<12.5 PBA. The vast majority did not have SA due to any of the diagnosis categories.

**Table 2:** Frequencies and proportions of sociodemographic characteristics, work-related factors, and SA diagnoses in 2019, for all and stratified by sex

Factors 2019	----------- All ------------		------- Women -------		--------- Men ---------	
	*n*	%	*n*	%	*n*	%
**Total**	1 347 778	100.00	633 194	100.00	714 584	100.00
Women	633 194	46.98				
Men	714 584	53.02				
**Age group (years)**						
19–24	52 809	3.92	29 976	4.73	22 833	3.20
25–34	310 459	23.03	153 168	24.19	157 291	22.01
35–44	348 994	25.89	159 960	25.26	189 034	26.45
45–54	371 812	27.59	170 287	26.89	201 525	28.20
55–64	237 834	17.65	108 153	17.08	129 681	18.15
65–68	25 495	1.89	11 497	1.82	13 998	1.96
**Level of education**						
Elementary (< 10 years)*	50 839	3.77	16 922	2.67	33 917	4.75
High school (10–12 years)	502 078	37.25	238 272	37.63	263 806	36.92
University (> 12 years)	794 861	58.98	378 000	59.70	416 861	58.34
**Country of birth**						
Sweden	1 162 038	86.22	538 899	85.11	623 139	87.20
Other Nordic countries	23 977	1.78	13 380	2.11	10 597	1.48
Other EU27 countries	41 132	3.05	20 121	3.18	21 011	2.94
Rest of the world*	120 631	8.95	60 794	9.60	59 837	8.37
**Family situation**						
Partner†, no children at home	266 315	19.76	124 542	19.67	141 763	19.84
Partner†, children at home	525 949	39.02	235 032	37.12	290 917	40.71
Single, no children at home*	492 311	36.53	229 827	36.30	262 484	36.73
Single, children at home	63 203	4.69	43 783	6.91	19 420	2.72
**Type of living area**						
Rural area	166 057	12.32	79 180	12.50	86 877	12.16
Town and suburb	501 119	37.18	229 842	36.30	271 277	37.96
City	680 602	50.50	324 172	51.20	356 430	49.88
**Company size**						
Micro (1–9)	148 417	11.01	70 075	11.07	78 342	10.96
Small (10–49)	255 642	18.97	114 815	18.13	140 827	19.71
Medium sized (50–249)	297 157	22.05	137 769	21.76	159 388	22.31
Large (≥ 250)	635 697	47.17	304 861	48.15	330 836	46.30
Info. is missing	10 865	0.81	5674	0.90	5191	0.73
**Branch of industry**						
Services	648 261	48.10	289 280	45.69	358 981	50.24
Manufacturing	248 457	18.43	80 194	12.66	168 263	23.55
Commerce and hospitality	114 177	8.47	60 711	9.59	53 466	7.48
Transport	49 751	3.69	19 150	3.02	30 601	4.28
Construction and installation	63 718	4.73	17 549	2.77	46 169	6.46
Care and education	214 167	15.89	160 844	25.40	53 323	7.46
Missing info.	9247	0.69	5466	0.86	3781	0.53
**Income‡**						
0,18-<4 PBA	94 304	7.00	65 234	10.30	29 070	4.07
4-<7.5 PBA	249 140	18.49	163 653	25.85	85 487	11.96
7.5-<10 PBA	347 148	25.76	181 348	28.64	165 800	23.20
10-<12.5 PBA	276 112	20.49	104 411	16.49	171 701	24.03
12.5-<15 PBA	159 389	11.83	48 192	7.61	111 197	15.56
15-<17.5 PBA	78 499	5.82	22 201	3.51	56 298	7.88
≥ 17.5 PBA	115 614	8.58	29 770	4.70	85 844	12.01
**SA diagnoses in 2019**						
**Depression**						
Yes	8055	0.60	5236	0.83	2819	0.39
No	1 339 723	99.40	627 958	99.17	711 765	99.61
**Anxiety**						
Yes	5329	0.40	3593	0.57	1736	0.24
No	1 342 449	99.60	629 601	99.43	712 848	99.76
**Exhaustion diagnosis**						
Yes	11 426	0.85	8093	1.28	3333	0.47
No	1 336 352	99.15	625 101	98.72	711 251	99.53
**Other stress-related diagnosis**						
Yes	12 784	0.95	9319	1.47	3465	0.48
No	1 334 994	99.05	623 875	98.53	711 119	99.52
**Other mental diagnosis**						
Yes	2126	0.16	1415	0.22	711	0.10
No	1 345 652	99.84	631 779	99.78	713 873	99.90
**Musculoskeletal diagnosis**						
Yes	15 636	1.16	10 045	1.59	5591	0.78
No	1 332 142	98.84	623 149	98.41	708 993	99.22
**Cancer**						
Yes	4666	0.35	3041	0.48	1625	0.23
No	1 343 112	99.65	630 153	99.52	712 959	99.77
**CVD**						
Yes	3131	0.23	1050	0.17	2081	0.29
No	1 344 647	99.77	632 144	99.83	712 503	99.71
**Injury**						
Yes	9679	0.72	5330	0.84	4349	0.61
No	1 338 099	99.28	627 864	99.16	710 235	99.39
**COVID-like diagnosis**						
Yes	4173	0.31	2832	0.45	1341	0.19
No	1 343 605	99.69	630 362	99.55	713 243	99.81
Other somatic diagnosis*						
Yes	16 171	1.20	10 923	1.73	5248	0.73
No	1 331 607	98.80	622 271	98.27	709 336	99.27
**Number of SA days in 2019**						
0	1 243 549	92.27	563 330	88.97	680 219	95.19
3.75 - <15	7525	0.56	5265	0.83	2260	0.32
15 - <31	27 337	2.03	18 006	2.84	9331	1.31
31 - <91	40 844	3.03	27 180	4.29	13 664	1.91
91 - <181	17 371	1.29	11 784	1.86	5587	0.78

Table note: * including cases where information was missing, † married/cohabitant, ‡ from work and work-related activities

**Fig. 1:**
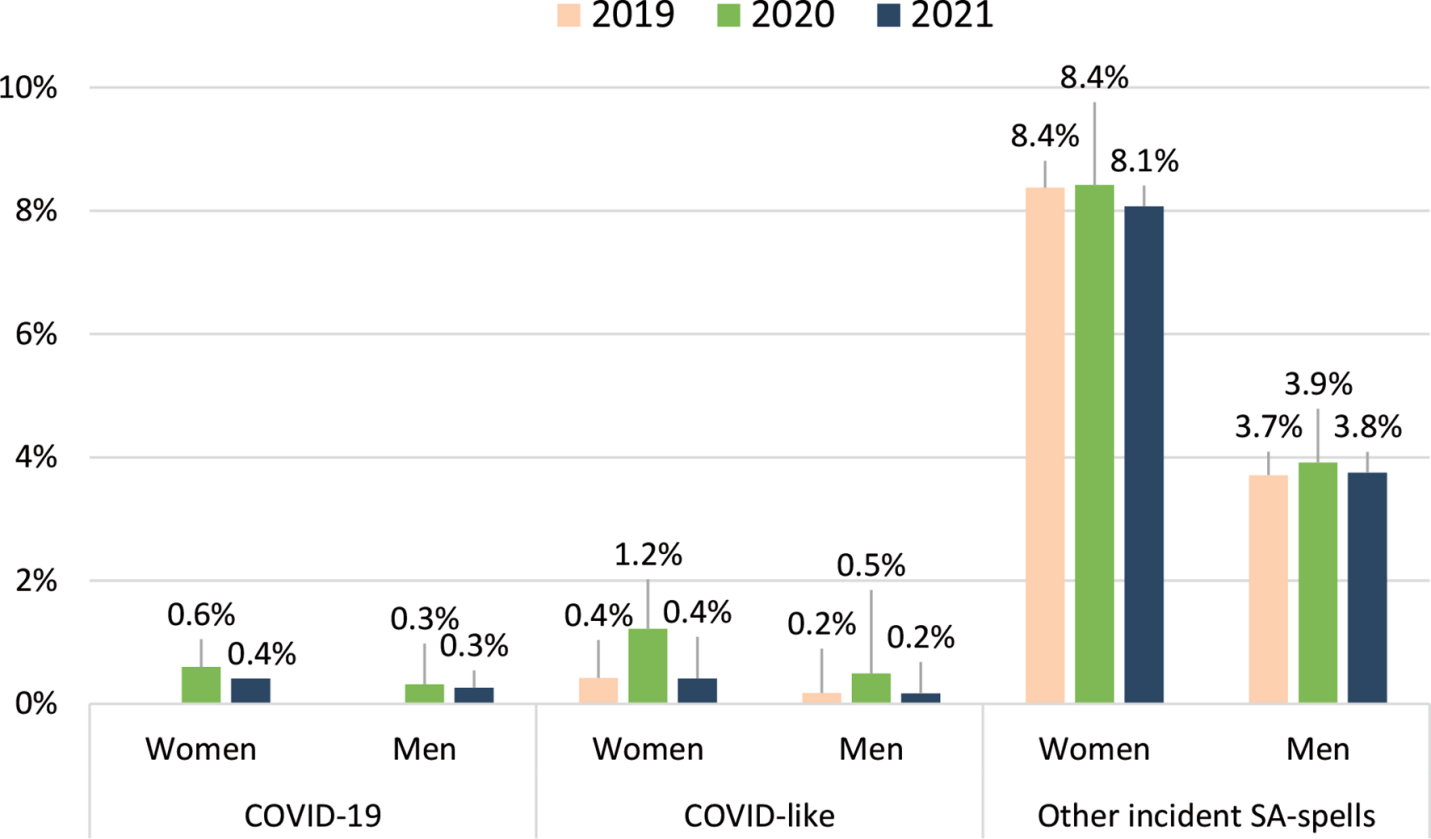
Proportion (%) of women and men with at least one incident SA-spell due to COVID-19 or due to a COVID-like diagnosis, as well as proportion (%) of women and men with other incident SA-spells (i.e., all other incident SA-spells) in 2019, 2020, and 2021, respectively

Figure 1 shows the proportions of individuals with incident SA due to COVID-19, COVID-like diagnoses, or all other SA, respectively. Note that individuals could belong to more than one category, if they had had SA spells with different diagnoses. The proportions of individuals with SA due to COVID-19 or COVID-like diagnoses were very low, at less than 1% among both women and men in all three years, except for women in 2020, where 1.2% had SA due to COVID-like diagnoses. In comparison, the proportions of individuals with SA due to any other diagnosis were higher: 8.4% among women during 2019 and 2020 and slightly lower at 8.1% in 2021. For men, the corresponding proportions were 3.7% in 2019, rising slightly to 3.9% in 2020 before decreasing to 3.8% in 2021. The absolute majority of the individuals had no incident SA at all (93.8% in 2019; 92.9% in 2020; 93.7% in 2021, data not shown in figure). In the study cohort, 18 women and 53 men died due to COVID-19 in 2020. In 2021, the corresponding numbers were 15 women and 64 men (not shown in figure).

In Supplementary Fig. 1, the proportions of individuals with all-cause incident SA categorized by branches of industry are displayed. These proportions were relatively similar across all branches of industry, except for care and education, which already in 2019 had a higher proportion of individuals with SA, as well as larger proportional increases in 2020 and 2021.

**Fig. 2:**
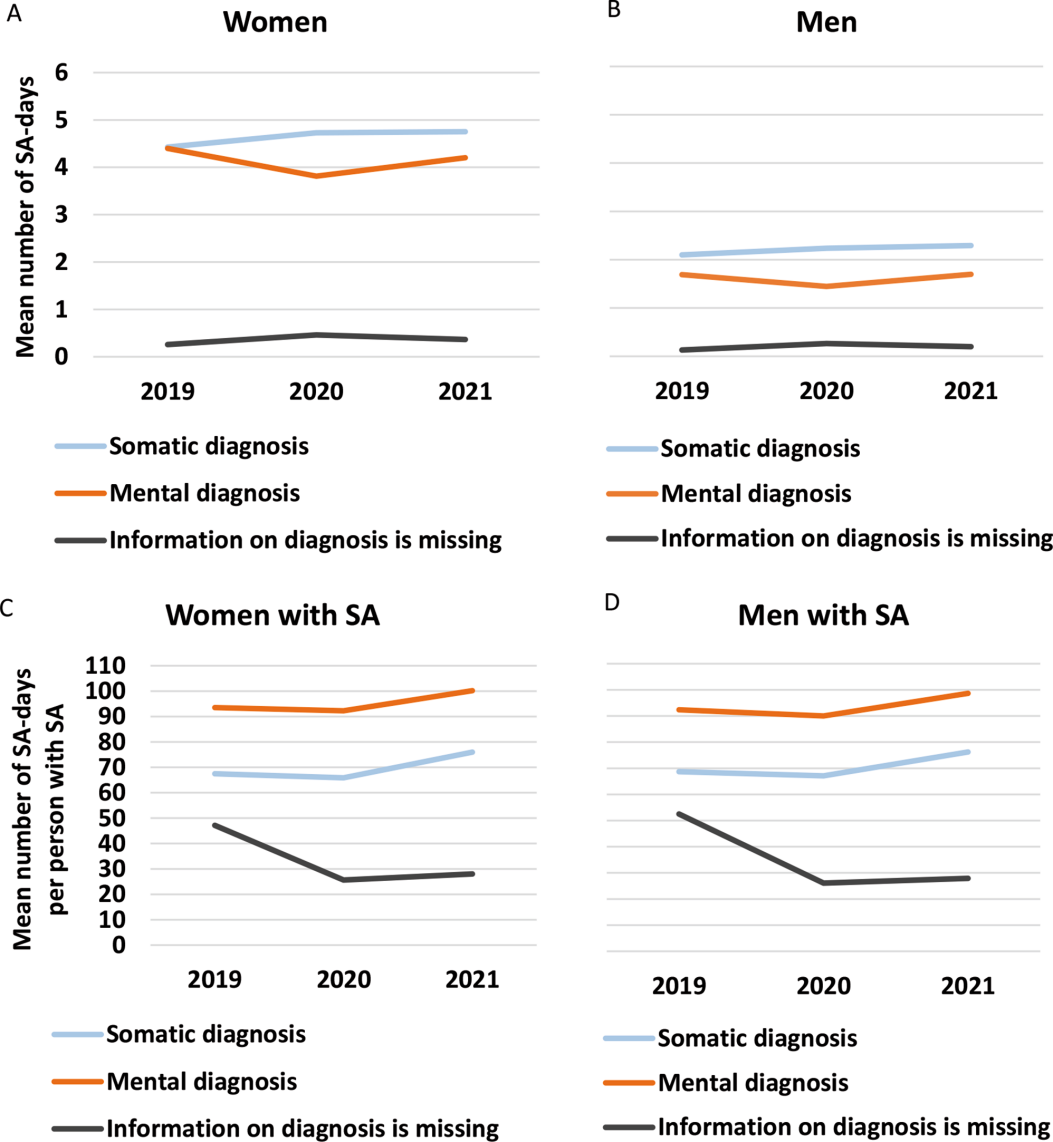
Mean number of annual sickness absence (SA) days per person among all women in the cohort (panel **A**) and among all men in the cohort (panel **B**) as well as among all women with SA the respective year (panel **C**) and among all men with SA the respective year (panel **D**) due to three diagnosis groups (somatic diagnoses, mental diagnoses, and where information on diagnosis is missing)

In Fig. 2, the mean numbers of annual SA net days due to somatic and mental diagnoses as well as for SA spells where information on diagnosis was missing, are displayed. In panel A, all women in the cohort are included, in panel B, all the men. In panels C and D, only the women and men who actually had some SA the respective year are included, thus the denominators are smaller. Considering panels A and B, the mean number of SA days with mental diagnoses decreased between 2019 and 2020 and increased between 2020 and 2021 for both women and men, while the mean number of SA days with somatic diagnoses increased each year between 2019 and 2021. Somatic SA diagnoses accounted for slightly more days than mental diagnoses. The mean number of SA days for which there was no information on diagnosis increased slightly in 2020.

When instead considering only individuals with SA in the respective diagnosis category during a given year (panels C and D), their mean numbers of SA days due to both mental and somatic diagnoses decreased slightly between 2019 and 2020, indicating that SA spells became shorter, however, the mean number of days increased quite substantially between 2020 and 2021, indicating longer SA. The mean number of SA days due to mental diagnoses was higher than that due to somatic diagnoses. The mean number of SA days for which there was no information on diagnosis became distinctly fewer.

**Fig. 3:**
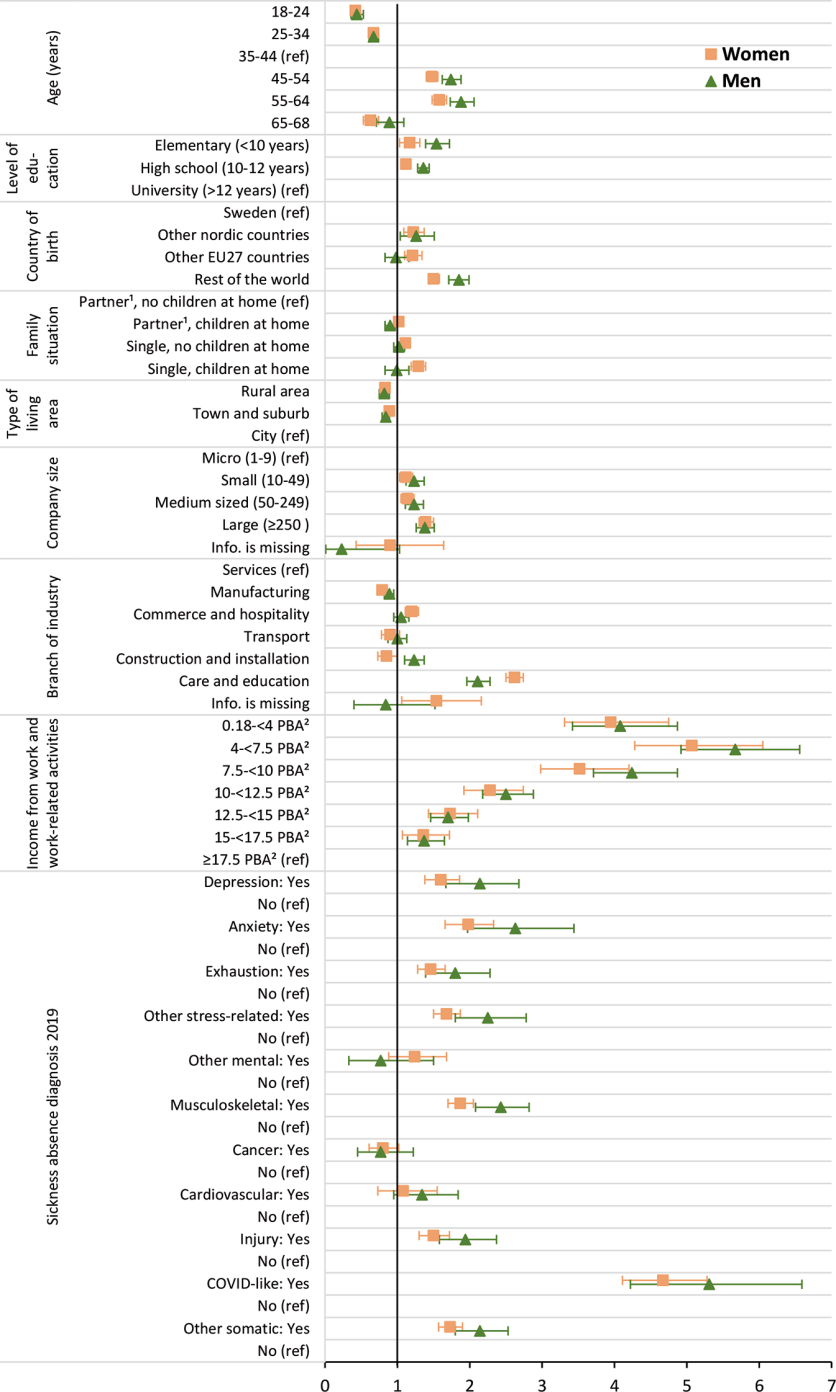
Adjusted odds ratios (OR) with 95% confidence intervals (CI) for having had at least one incident sickness absence spell due to COVID-19/COVID-like diagnosis in 2020 as outcome; including socio-demographic and work-related characteristics as well as sickness absence diagnoses in 2019 as factors; for women (ORs depicted as orange squares) and men (ORs depicted as green triangles). ^1^ “Partner” denotes married or cohabiting. ^2^ “PBA” is short for “price base amount”

Figure 3 and Supplementary Table 1 show odds ratios (OR) and 95% confidence intervals (CI) from crude and adjusted logistic regression models exploring the association between having at least one incident SA spell due to COVID-19 or COVID-like diagnoses in 2020 and a variety of relevant factors measured in 2019 (sociodemographic, work-related, as well as factors pertaining to prior SA diagnoses). In Supplementary Table 1, the proportions of individuals with SA due to COVID-19 or COVID-like diagnoses are shown for all factors (at their respective categories) included in the regression models.

The ORs for most sociodemographic factors were either non-significant, or relatively small, rarely above 1.50 or below 0.67 in magnitude. However, individuals born outside the EU had OR of 1.85 (95% CI 1.71–1.99) for men and 1.50 (95% CI 1.43–1.58) for women. Odds ratios were often higher for men, indicating larger differences between men than women. A notable exception was for those who worked in care and education, where women had an OR of 2.62 (95% CI 2.50–2.74) and men had an OR of 2.11 (1.96–2.28) compared to those working in services.

Large differences were seen for income, where those earning less than 10 PBA had markedly elevated ORs compared to the reference group, i.e., the group with the highest income earning 17.5 PBA or more. A gradient was seen with respect to income; individuals with lower incomes had higher ORs for SA due to COVID-19 or COVID-like diagnoses. However, the lowest income group 0.18-<4 PBA had slightly lower ORs (among women, 3.95, 95% CI 3.31–4.75; among men 4.08, 95% CI 3.42–4.87) than the second lowest group, 4-<7.5 PBA (among women 5.07, 95% CI 4.28–6.05; among men 5.67, 95% CI 4.92–6.56).

Prior SA (i.e., SA in 2019) due to COVID-like diagnoses was the factor with the strongest association with incident SA due to COVID-19 or COVID-like diagnoses in 2020, with ORs of 4.67 (95% CI 4.11–5.28) for women and 5.31 (95% CI 4.22–6.59) for men. Prior SA due to most mental diagnoses was associated with higher ORs for SA due to COVID-19 or COVID-like disorders of between 1.5 and 2.5 compared to those without the respective diagnosis (e.g., depression, 1.60, 95% CI 1.38–1.86 for women, 2.14, 95% CI 1.67–2.68 for men, or anxiety 1.98, 95% CI 1.66–2.33 for women, 2.63, 95% CI 1.97–3.44 for men), although the associations were non-significant for the group other mental diagnoses. For prior SA due to musculoskeletal diagnoses (1.87, 95% CI 1.70–2.05 for women; 2.43, 95% CI 2.08–2.82 for men), injury diagnoses (1.50, 95% CI 1.30–1.72 among women, 1.94, 95% CI 1.58–2.37 among men) and the group other somatic diagnoses (1.73, 95% CI 1.57–1.90 for women, 2.14, 95% CI 1.80–2.53 for men), the ORs were also of comparable magnitudes as for the mental diagnoses, whereas they were non-significant for prior SA due to cancer and cardiovascular diagnoses.

Sensitivity analyses for the logistic regression analyses were conducted, and the results are shown in Supplementary Table 2. For the sensitivity analyses the outcome was redefined as having had at least one SA spell >14 days due to COVID-19 in 2020, i.e., the criterion for qualifying for the outcome no longer included SA spells due to COVID-like diagnoses. Overall, very similar results compared to the main analysis were obtained in the sensitivity analyses. Prior SA due to COVID-like diagnoses was still among the factors that were most strongly associated with SA due to COVID-19, although the estimates were lower than in the main analysis (2.45, 95% CI 1.86-3.16 for women, 2.23, 95% CI 1.28-3.60 for men).

## Discussion

In this population-based prospective cohort study of privately employed white-collar workers, we found that the vast majority (94%) had no incident sickness absence (SA) spells > 14 days in 2019 and 2021, and 93% had no such incident SA in 2020. Moreover, prior SA due to COVID-like diagnoses and low income were the factors most strongly associated with SA due to COVID-19 or COVID-like diagnoses in 2020.

We found, in general, only small changes in SA for these first two years of the COVID-19 pandemic compared to in the previous year, both regarding proportions of individuals with an incident SA spell and the mean number of SA days per person. A very small proportion of the study population had SA due to COVID-19 or COVID-like diagnoses, at the most approx. 2% (among women in 2020). The absolute majority of individuals with SA had SA due to other diagnoses than COVID-19 or COVID-like diagnoses. Since COVID-19 often led to SA spells of shorter duration than many other diagnoses [5, 22, 23], it is probable that the results would have been different if we had been able to also include shorter SA spells (< 15 days). We have only found two other comparable studies, regarding occupational groups and SA measures. One covers privately employed blue-collar workers in the retail and wholesale industry in Sweden [24] and their proportions of individuals with SA in general and due to COVID-19 and COVID-like diagnoses specifically were slightly higher than those found in our study. The other study included healthcare workers in Norway; their SA rates more than doubled during certain months of the pandemic compared to the corresponding months in 2017–2019 [20]. The increases seen in our study are thus smaller than those seen in these two studies. That the proportion of individuals with SA > 14 days increased to such a small extent in our study might suggest that many in this group were able to work from home, in line with the Swedish strategy to handle the pandemic. However, to what extent this actually was the case cannot be determined from our study. Furthermore, we only have information on SA spells > 14 days, something most individuals with COVID-19 did not need. A study from Sweden on those who were aged 18–64, employed or self-employed, and had a positive polymerase chain reaction (PCR) test for SARS-CoV-2 during the period 1 January 2020 until 31 August 2021, found that 5.7% of the individuals had an incident SA spell > 14 days in the period 7 days before to 30 days after the test, meaning that the vast majority of those who had a COVID-19 infection did not require SA > 14 days due to the infection [25]. However, we cannot compare our results with those from that study, as we did not have any information on COVID-19 infection status, and, therefore, no information on the proportion of our study population who had COVID-19 without requiring SA > 14 days due to that diagnosis.

While the proportion of individuals with SA > 14 days slightly increased overall, the mean number of SA days per person with such SA decreased, indicating that also spells exceeding 14 days tended to be of shorter duration than previously. This was also reported by the Social Insurance Agency for the general working population [5, 22]. However, in our cohort this was not the case in 2021, rather the mean number of SA days per person with SA increased, both regarding mental and somatic SA diagnoses. It might be that accessing healthcare and rehabilitation became more difficult during the pandemic which may have led to delays in treatment, i.e., individuals who would normally have sought healthcare either refrained from doing so or sought healthcare later in the disease trajectory when the symptoms were more severe, or faced longer waiting times for treatments [5]. Investigations into healthcare consumption during the pandemic found that both phenomena were observed [26, 27]. During 2020, both the number of patients waiting to be seen by healthcare providers and the number of patients actually seen by specialists decreased by 18% compared to the mean in 2017–2019, meaning that the queues for specialist healthcare remained fairly stable. This then changed in 2021, when the number of patients waiting to be seen increased by 40% compared to 2020, while the number of patients actually seen only increased by 15%, meaning that the waiting times for healthcare increased substantially during 2021. Psychiatric care was one of the specialist branches where the waiting times increased the most. At the same time, approximately half of the respondents to a questionnaire sent to a random sample of the population in Sweden reported that they had either refrained from seeking healthcare or waited to seek healthcare due to the pandemic [26, 27]. This could explain the lower prevalence of SA due to mental diagnoses in the first year of the pandemic, 2020. Additionally, the increase in length of SA due to both somatic and mental diagnoses in 2021 may be linked to such delays. If individuals postponed seeking healthcare or had to wait for assessment, treatment, and rehabilitation, their work incapacity might have been prolonged. This warrants further investigations in future studies.

In terms of sociodemographic factors, the associations observed between these factors and SA due to COVID-like diagnoses aligned with findings from previous studies investigating SA across various diagnoses [10, 13, 17]. For instance, women exhibited a higher likelihood of having SA compared to men, despite men having a higher risk for severe COVID-19 and COVID-19 mortality in all age groups, including the ones studied here [1, 28]. Even in our study, of the few that died due to COVID-19 during the study period, the proportion of men was higher.

Furthermore, there were discernible gradients concerning education and age, where individuals with lower educational attainment and those in higher age brackets were more likely to have SA, which is also in line with previous findings [17, 18]. Similarly, those born in the rest of the world were also more likely to have SA, in line with previous findings [10, 11, 13, 17]. Another contributing factors could be that the incidence and mortality of COVID-19 were higher among foreign-born, particularly those born in the rest of the world [29, 30].

Previous studies of all-cause SA have also found an income gradient where the risk of SA is lower in higher income groups [31]. When solely comparing the two lowest income groups in our study we did not see this gradient, i.e., the lowest income group had slightly lower odds ratios of SA due to COVID-19 and COVID-like diagnoses than the second lowest income group, although they had overlapping confidence intervals among women. However, this anomaly may be attributed to more varied and less stable employment patterns in the lowest income group, which potentially means that they were either not eligible for or had less need of SA due to COVID-19 or other ailments. Additionally, this observation is possibly influenced by the exceptionally low-income threshold employed for inclusion in our study.

Our results regarding branch of industry showed that healthcare and education was by far the branch of industry with the highest OR for SA due to COVID-19 or COVID-like diagnoses among the privately employed white-collar workers. This is in line with a Swedish report, that found that the incidence of COVID-19 was higher in these branches of industry than in others, including other occupations with contacts with the public [32], Another Swedish report found that those branches had a large increase of SA during the pandemic [33]. However, none of these studies differentiated by type of worker or type of employer (public or private). Our results show that this was also the case for SA among privately employed white-collar workers, although the magnitude of the difference was slightly smaller in our study than the differences in incidence of COVID-19 when the total labour market was considered. It is possible that individuals in these branches of industry had less opportunities to work from home, which has been found in the US [34, 35], Italy [36], and Germany [37], although to our knowledge this has not been studied in Sweden.

Although mental diagnoses represent a common cause for SA, when compared to somatic diagnoses, somatic diagnoses were still responsible for more SA days both before and during the pandemic, and the difference increased during the pandemic. We found that mental SA diagnoses decreased in both the number of days per person, and the number of days per person with SA due to mental diagnoses, indicating both that fewer individuals had SA due to mental diagnoses and that the length of SA due to mental diagnoses became shorter in 2020. Research into the effects of the pandemic on mental health is inconclusive, with systematic reviews finding that the prevalence of anxiety and depression as well as symptoms of depression increased slightly during the pandemic (although in some studies, only in the first months or during lockdowns) [38–40]. The results regarding general mental health were also inconclusive, with one review with a meta-analysis finding no significant effect [40] and another a slight decrease during the pandemic [39]. However, none of these reviews have considered SA: while mental health might overall have deteriorated, it is possible that working from home enabled people with various mental health conditions to remain in work [41]. While there has been some research on SA due to mental diagnoses on healthcare staff during the pandemic [8, 42, 43], it is unlikely that those are generalisable to other occupations and branches of industry. More studies are needed on how SA due to both mental and somatic diagnoses (other than COVID-19) developed during the pandemic.

Those with prior SA due to depression, anxiety, exhaustion disorder, or other stress-related disorders had higher odds of having SA due to COVID-19 or COVID-like diagnoses than those who did not have such prior SA, whereas the association was non-significant for those with other mental diagnoses. Since SA spells in these diagnoses often become long [44], it is possible that they were still on SA due to the mental diagnosis, and thus were not eligible for new SA. Some studies have shown that individuals with mental diagnoses had higher rates of COVID-19 infection during the pandemic, as well as higher rates of hospitalisation and mortality when infected, than those without [45–47], although the difference between those with mental diagnoses and those with somatic diagnoses was in some cases non-significant [48]. The results from our study indicate that SA due to COVID-19 or COVID-like diagnoses—where the disease became severe enough to limit a person’s work capacity for more than two weeks—also follows this pattern.

For those with prior SA due to cancer or to cardiovascular diagnoses, the association with SA due to COVID-19 or COVID-like diagnoses was non-significant. This was surprising, since both malignant cancer and certain forms of CVD have been identified as risk factors for severe COVID-19, and there were special recommendations regarding people with such conditions, both to the individual and to healthcare [49]. It is possible that these individuals had SA due to other diagnoses, such as the same diagnosis as their previous SA. It is also possible that these individuals were more cautious in their social contacts and stringent in their self-isolation, based on their status as having a risk factor of severe COVID-19, and therefore less exposed to COVID-19 infection. Some research has suggested that people with pre-existing morbidity were more likely to comply with social distancing and isolation regulations or guidelines [50].

We did not consider the white-collar workers’ actual morbidity in this article. Most individuals with some kind of disease or injury are not on SA, since their disease or injury does not affect their work capacity to the extent that they need to be on SA [51–53]. This seemed to also be the case for COVID-19, where one study in Sweden found that 5.7% of those with confirmed COVID-19 were on SA > 14 days starting within 7 days before and 30 days after their COVID-19 test [25]. More knowledge is needed on the possibilities to remain in paid work with different kinds of morbidity as well as on the rates and patterns of SA due to specific diagnoses among privately employed white-collar workers.

### Strengths and limitations

The principal strength of this study is its use of a comprehensive, population-based cohort encompassing all 1 347 778 eligible individuals in Sweden. This approach ensures that the study is not reliant on a sample and it provides a sufficiently large population for conducting subgroup analyses. Additionally, each individual could be followed up from inclusion until death, emigration, or the end of the follow-up period, resulting in no drop-outs. Other significant strengths include the utilization of linked microdata from three high-quality nationwide administrative registers and the absence of self-reported data, thereby eliminating the potential for recall bias [54, 55]. It is also a strength that we used measures of both the incidence and length of SA, something previous research has shown the necessity of [10, 56].

The study also has some limitations. Its exploratory and observational nature precludes the establishment of causal inferences. That SA spells shorter than 15 days were not included, can be seen as both as a strength and a limitation. For many people of working ages, SA due to COVID-19 was often mild and of shorter duration [22, 23]. Thus, the SA examined here represents only the more severe cases of COVID-19 or COVID-like diseases, meaning those that led to a reduction of work capacity for at least 15 days. Furthermore, even if we had had information on shorter SA spells, information on diagnosis would not have been available until day 8 at the earliest, as SA spells shorter than 8 days did not need to be certified by a physician (during parts of the pandemic, this was extended to 21 days).

Moreover, we only had information on the first main SA diagnoses, which might have led to an underestimation of SA spells due to COVID-19, if COVID-19 was a secondary diagnosis or if the individuals received other diagnoses before their diagnosis of COVID-19. This was one of the reasons we also included the COVID-like diagnoses in our analyses.

Another limitation pertains to the reliability of COVID-19 diagnoses. In the early stages of the pandemic, before widespread testing, individuals with suspected COVID-19 were often assigned different diagnoses on medical certificates. To address this, we followed the Social Insurance Agency’s practice of including certain infectious, respiratory, and symptom-based diagnoses which we refer to with the term “COVID-like” (whereas the Social Insurance Agency uses the term “COVID-related diagnoses”). This might have led to misclassifications, as not all the SA spells included in this category were due to COVID-19. Some of the diagnoses included in this category were, e.g., Viral and other specified intestinal infections, Viral infection of unspecified site, Acute nasopharyngitis [common cold], Influenza due to unidentified influenza virus, Acute bronchitis, Asthma, Cough, Abnormalities of breathing, Rash and other nonspecific skin eruption, Fever of other and unknown origin, and Malaise and fatigue. Some individuals had SA with these diagnoses already in 2019, when it is highly unlikely it was COVID-19, and they might have had SA due to the same condition in 2020 rather than due to COVID-19. This might, thus, have led to an overestimation of COVID-like SA, as well as an overestimation of the association between prior SA in COVID-like diagnoses and incident SA due to COVID-19 or COVID-like diagnoses in 2020, which is somewhat supported by the associations being lower in the sensitivity analyses. However, if we had not included these diagnoses in our analyses, we might have underestimated SA that was likely due to COVID-19. Our descriptive results showed that the increase in the proportion of individuals with SA due to COVID-like diagnoses between 2019 and 2020 was of the same magnitude as the proportion of individuals with SA due to a COVID-19 diagnosis in 2020: not including these diagnoses might thus have led to missing approximately half of the individuals with SA due to suspected COVID-19. However, as previously stated, not all those were necessarily COVID-19; it is plausible that some individuals extended their SA spells due to other, similar, conditions in order not to infect their co-workers. Unfortunately, we have no way of knowing which of the SA spells with a COVID-like diagnosis were actually due to a COVID-19 infection and which were due to other diseases with similar symptoms. Conversely, it is possible that there were additional diagnoses that were used for symptoms of COVID-19 in the beginning of the pandemic that were not classified by the Social Insurance Agency as “COVID-related”, potentially leading to an underestimation of COVID-like SA. Given COVID-19’s diverse impact on various organs and its wide range of symptoms [57], the category of COVID-like diagnoses could have been broadened to include other potential diagnoses comprising symptoms like COVID-19 when the status was unconfirmed.

As in most studies, additional information that would have been useful was not available. For instance, we had no information on the workers’ possibilities to work from home, which would have been informative when interpreting differences in SA.

## Conclusion

In this study of sickness absence (SA) among privately employed white-collar workers, we found that only small changes of SA occurred during the COVID-19 pandemic compared to the previous year, and that SA due to COVID-19 was a small part of all SA during this period. We also found that the factors associated with SA due to COVID-19 or COVID-like diagnoses to some degree corresponded with the factors associated with SA prior to the pandemic, indicating that those who were more likely to have SA due to other diagnoses were also more likely to have SA due to COVID-19 or COVID-like diagnoses.

## Electronic supplementary material

Below is the link to the electronic supplementary material.

Supplementary Material 1

## Data Availability

The used data cannot be made publicly available due to privacy regulations. According to the General Data Protection Regulation, the Swedish law SFS 2018:218, the Swedish Data Protection Act, the Swedish Ethical Review Act, and the Public Access to Information and Secrecy Act, these types of sensitive data can only be made available for specific purposes that meets the criteria for access to this type of sensitive and confidential data as determined by a legal review. Professor Kristina Alexanderson (Kristina.alexanderson@ki.se) can be contacted regarding the data.
