# Supplementary material for: Sickness absence among privately employed white-collar workers during the COVID-19 pandemic; a prospective cohort study
Source: BMC Public Health. 2025 Feb 10;25:548. doi: 10.1186/s12889-025-21566-1 (PMC11812151; doi:10.1186/s12889-025-21566-1)
Supplement: Supplementary file 1 — Supplementary Material 1 [file 12889_2025_21566_MOESM1_ESM.pdf]

Supplementary Materials

Sickness absence among privately employed white-collar workers during the COVID-19 pandemic; a prospective cohort study

Kristin Farrants, Mira Müller, Kristina Alexanderson

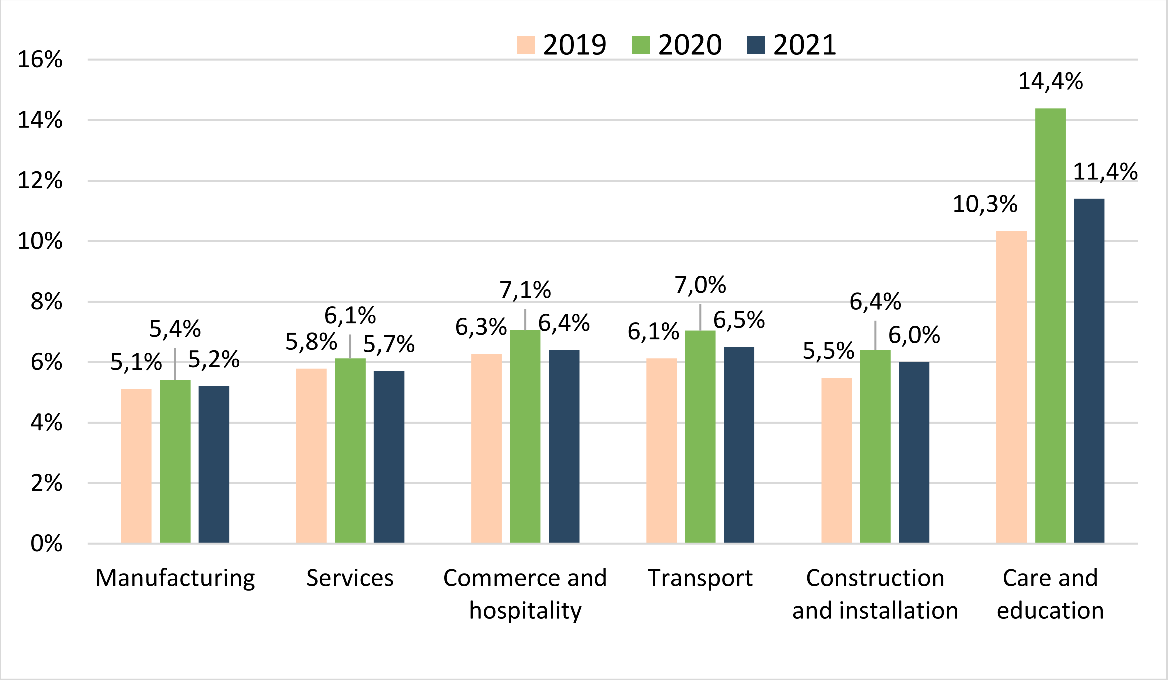

Supplementary Figure 1: Proportion (%) of people with at least one incident SA-spell per year and branch of industry.

Supplementary Table 1: Unadjusted (crude) (columns 2-4) and adjusted (columns 5-7) odds ratios (OR) with 95% confidence interval (CI) for having had at least one incident SA-spell due to COVID-19/COVID-like diagnosis in 2020 as outcome; including socio-demographic and work-related characteristics as well as SA-diagnoses in 2019 as factors; for all and stratified by sex. Frequencies (n) and proportion (%) (columns 8-10) of people in each category with the outcome of having had at least one incident SA-spell due to COVID-19/COVID-like diagnosis in 2020, for all and stratified by sex. (Reported proportions are calculated based on all people in each category, i.e., for the total the denominator was n= 1 347 778).

	**Crude**			**Adjusted**			**Frequencies**		
	**All**	**Women**	**Men**	**All**	**Women**	**Men**	**All**	**Women**	**Men**
**Factors 2019**	**OR(95 % CI)**	**OR(95 % CI)**	**OR(95 % CI)**	**OR(95 % CI)**	**OR(95 % CI)**	**OR(95 % CI)**	**n(%)**	**n(%)**	**n(%)**
**Total**							14 409(1.07)	9326(1.47)	5083(0.71)
**Sex**									
Woman	Ref.	-	-	Ref.	-	-	9326(1.47)	-	-
Man	0.45(0.43-0.46)	-	-	0.79(0.76-0.82)	-	-	5083(0.71)	-	-
**Age group (years)**									
19-24	0.68(0.61-0.75)	0.55(0.49-0.62)	0.83(0.64-1.05)	0.43(0.38-0.48)	0.42(0.36-0.47)	0.44(0.36-0.53)	328(0.62)	221(0.74)	107(0.47)
25-34	0.76(0.71-0.82)	0.74(0.70-0.78)	0.80(0.71-0.91)	0.67(0.64-0.71)	0.67(0.63-0.71)	0.67(0.61-0.74)	2318(0.75)	1505(0.98)	813(0.52)
35-44	Ref.	Ref.	Ref.	Ref.	Ref.	Ref.	3184(0.91)	2117(1.32)	1067(0.56)
45-54	1.39(1.34-1.45)	1.36(1.29-1.43)	1.52(1.38-1.68)	1.56(1.49-1.63)	1.48(1.40-1.56)	1.74(1.62-1.88)	4702(1.26)	3037(1.78)	1665(0.83)
55-64	1.71(1.63-1.78)	1.64(1.56-1.73)	1.79(1.62-1.98)	1.68(1.59-1.77)	1.58(1.48-1.68)	1.88(1.73-2.06)	3673(1.54)	2329(2.15)	1344(1.04)
65-68	0.77(0.65-0.91)	0.88(0.77-0.99)	0.40(0.25-0.61)	0.72(0.63-0.82)	0.63(0.53-0.74)	0.89(0.71-1.09)	204(0.80)	117(1.02)	87(0.62)
**Level of education**									
Elementary (<10 years)*	1.35(1.25-1.46)	1.18(1.04-1.32)	2.04(1.77-2.34)	1.35(1.25-1.46)	1.17(1.03-1.31)	1.54(1.39-1.72)	603(1.19)	247(1.46)	356(1.05)
High school (10-12 years)	1.53(1.48-1.58)	1.45(1.40-1.51)	1.71(1.59-1.84)	1.19(1.15-1.23)	1.12(1.08-1.17)	1.36(1.28-1.44)	7045(1.40)	4324(1.81)	2721(1.03)
University (>12 years)	Ref.	Ref.	Ref.	Ref.	Ref.	Ref.	6761(0.85)	4755(1.26)	2006(0.48)

**Country of birth**									
Sweden	Ref.	Ref.	Ref.	Ref.	Ref.	Ref.	11 107(0.96)	7111(1.32)	3996(0.64)
Other Nordic countries	1.48(1.32-1.65)	1.60(1.45-1.76)	1.48(1.14-1.89)	1.23(1.12-1.36)	1.22(1.09-1.37)	1.26(1.04-1.51)	365(1.52)	259(1.94)	106(1.00)
Other EU27 countries	1.22(1.12-1.33)	1.31(1.19-1.45)	1.08(0.87-1.32)	1.14(1.05-1.24)	1.21(1.10-1.34)	0.98(0.83-1.16)	479(1.16)	347(1.72)	132(0.63)
Rest of the world*	2.04(1.94-2.14)	2.16(2.08-2.25)	1.92(1.74-2.12)	1.60(1.53-1.67)	1.50(1.43-1.58)	1.85(1.71-1.99)	2458(2.04)	1609(2.65)	849(1.42)
**Family situation**									
Partner†, no children at home	Ref.	Ref.	Ref.	Ref.	Ref.	Ref.	3330(1.25)	2119(1.70)	1211(0.85)
Partner†, children at home	0.73(0.70-0.77)	0.70(0.67-0.73)	0.74(0.67-0.81)	0.98(0.94-1.03)	1.02(0.96-1.08)	0.90(0.83-0.97)	4652(0.88)	2945(1.25)	1706(0.59)
Single, no children at home*	0.85(0.81-0.90)	0.87(0.83-0.90)	0.82(0.75-0.90)	1.07(1.03-1.12)	1.11(1.06-1.17)	1.02(0.95-1.10)	5358(1.09)	3357(1.46)	2001(0.76)
Single, children at home	1.22(1.13-1.31)	1.36(1.27-1.45)	1.09(0.89-1.33)	1.23(1.14-1.31)	1.29(1.19-1.39)	0.99(0.83-1.16)	1069(1.69)	905(2.07)	164(0.84)
**Type of living area**									
Rural area	1.21(1.13-1.29)	1.02(0.96-1.08)	1.29(1.15-1.43)	0.83(0.79-0.87)	0.83(0.78-0.88)	0.82(0.75-0.89)	1844(1.11)	1195(1.51)	649(0.75)
Town and suburb	1.13(1.08-1.19)	0.98(0.94-1.02)	1.17(1.08-1.26)	0.87(0.84-0.90)	0.89(0.85-0.93)	0.84(0.79-0.89)	5158(1.03)	3335(1.45)	1823(0.67)
City	Ref.	Ref.	Ref.	Ref.	Ref.	Ref.	7408(1.09)	4796(1.48)	2612(0.73)
**Company size**									
Micro (1-9)	Ref.	Ref.	Ref.	Ref.	Ref.	Ref.	>1275(0.86)	>750(1.10)	>500(0.65)
Small (10-49)	1.09(1.02-1.15)	1.12(1.04-1.22)	1.01(0.88-1.16)	1.16(1.09-1.24)	1.12(1.03-1.21)	1.23(1.12-1.37)	2390(0.93)	1473(1.28)	917(0.65)
Medium sized (50-249)	1.13(0.98-1.29)	1.16(1.07-1.25)	1.10(1.04-1.17)	1.17(1.10-1.25)	1.14(1.05-1.23)	1.23(1.11-1.36)	2818(0.95)	1753(1.27)	1065(0.67)
Large (≥250)	1.43(1.36-1.52)	1.60(1.49-1.71)	1.20(1.06-1.36)	1.40(1.32-1.48)	1.39(1.30-1.50)	1.38(1.26-1.51)	7913(1.24)	5321(1.75)	2592(0.78)
Missing info.	0.93(0.45-1.70)	0.67(0.34-1.19)	1.09(0.27-2.85)	0.69(0.34-1.22)	0.90(0.43-1.64)	0.23(0.01-1.03)	<10(0.07)	<10(0.10)	<10(0.02)
**Branch of industry**									
Services	Ref.	Ref.	Ref.	Ref.	Ref.	Ref.	4619(0.71)	2554(0.88)	2065(0.58)
Manufacturing	0.95(0.89-1.02)	0.81(0.75-0.88)	1.02(0.93-1.12)	0.86(0.81-0.90)	0.79(0.73-0.86)	0.89(0.82-0.95)	1491(0.60)	575(0.72)	916(0.54)
Commerce and hospitality	1.15(1.06-1.25)	1.34(1.24-1.44)	1.10(0.95-1.26)	1.15(1.09-1.23)	1.20(1.11-1.29)	1.05(0.95-1.16)	1115(0.98)	715(1.18)	400(0.75)
Transport	1.38(1.23-1.54)	1.06(0.92-1.21)	1.45(1.23-1.69)	0.97(0.88-1.07)	0.90(0.78-1.03)	1(0.87-1.13)	402(0.81)	179(0.93)	223(0.73)
Construction and installation	1.09(0.97-1.22)	0.86(0.74-1.01)	1.23(1.07-1.42)	1.12(1.02-1.22)	0.85(0.73-1.00)	1.23(1.10-1.37)	475(0.75)	134(0.76)	341(0.74)

Care and education	2.25(2.13-2.37)	3.69(3.54-3.86)	1.92(1.72-2.15)	2.50(2.40-2.59)	2.62(2.50-2.74)	2.11(1.96-2.28)	6210(2.90)	5097(3.17)	1113(2.09)
Info. is missing	1.60(1.26-1.98)	1.48(1.19-1.82)	1.98(1.34-2.80)	1.30(0.94-1.75)	1.54(1.06-2.16)	0.84(0.40-1.52)	97(1.04)	72(1.32)	24(0.63)
**Income‡**									
0.18-<4 PBA	4.09(3.45-4.90)	4.98(4.45-5.57)	3.33(2.68-4.14)	4.21(3.75-4.74)	3.95(3.31-4.75)	4.08(3.42-4.87)	1275(1.35)	993(1.52)	282(0.97)
4-<7.5 PBA	6.50(5.51-7.73)	7.69(6.95-8.54)	4.21(3.54-5.04)	5.47(4.92-6.11)	5.07(4.28-6.05)	5.67(4.92-6.56)	5308(2.13)	3910(2.39)	1398(1.64)
7.5-<10 PBA	4.02(3.40-4.78)	4.62(4.17-5.14)	3.11(2.63-3.71)	3.91(3.52-4.35)	3.52(2.98-4.20)	4.24(3.71-4.87)	4437(1.28)	2710(1.49)	1727(1.04)
10-<12.5 PBA	2.43(2.05-2.92)	2.58(2.32-2.88)	2.29(1.93-2.73)	2.46(2.21-2.75)	2.28(1.92-2.74)	2.50(2.18-2.88)	2009(0.73)	953(0.91)	1056(0.62)
12.5-<15 PBA	1.75(1.44-2.13)	1.75(1.55-1.97)	1.69(1.40-2.05)	1.75(1.55-1.97)	1.73(1.43-2.11)	1.70(1.46-1.98)	765(0.48)	317(0.66)	448(0.40)
15-<17.5 PBA	1.37(1.19-1.59)	1.35(1.07-1.71)	1.36(1.08-1.71)	1.38(1.20-1.60)	1.36(1.07-1.72)	1.37(1.14-1.65)	297(0.38)	113(0.51)	184(0.33)
≥17.5 PBA	Ref.	Ref.	Ref.	Ref.	Ref.	Ref.	318(0.28)	112(0.38)	206(0.24)
**SA diagnosis 2019**									
**Depression**									
Yes	2.61(2.30-2.94)	2.03(1.75-2.35)	2.89(2.03-3.98)	1.74(1.53-1.98)	1.60(1.38-1.86)	2.14(1.67-2.68)	218(2.71)	153(2.92)	65(2.31)
No	Ref.	Ref.	Ref.	Ref.	Ref.	Ref.	14 191(1.06)	9173(1.46)	5018(0.71)
**Anxiety**									
Yes	2.81(1.77-4.21)	2.36(2.00-2.78)	3.07(2.66-3.52)	2.15(1.86-2.48)	1.98(1.66-2.33)	2.63(1.97-3.44)	169(3.17)	121(3.37)	48(2.76)
No	Ref.	Ref.	Ref.	Ref.	Ref.	Ref.	14 240(1.06)	9205(1.46)	5035(0.71)
**Exhaustion**									
Yes	2.14(1.91-2.40)	1.67(1.46-1.89)	3.59(2.68-4.69)	1.54(1.37-1.73)	1.46(1.28-1.66)	1.80(1.39-2.28)	255(2.23)	195(2.41)	60(1.80)
No	Ref.	Ref.	Ref.	Ref.	Ref.	Ref.	14 154(1.06)	9131(1.46)	5023(0.71)
**Other stress-related**									
Yes	2.62(1.88-3.55)	2.08(1.86-2.31)	2.71(2.46-2.99)	1.79(1.62-1.98)	1.68(1.50-1.87)	2.25(1.80-2.78)	356(2.78)	276(2.96)	80(2.31)
No	Ref.	Ref.	Ref.	Ref.	Ref.	Ref.	14 053(1.05)	9050(1.45)	5003(0.70)
**Other mental**									
Yes	1.73(1.28-2.28)	1.57(1.13-2.12)	1.95(0.77-3.96)	1.15(0.84-1.52)	1.24(0.88-1.68)	0.77(0.33-1.50)	38(1.79)	32(2.26)	<10(0.84)
No	Ref.	Ref.	Ref.	Ref.	Ref.	Ref.	14 371(1.07)	9294(1.47)	>5075(0.71)

**Musculoskeletal**									
Yes	3.82(3.54-4.12)	3.15(2.88-3.44)	3.60(2.87-4.44)	2(1.85-2.16)	1.87(1.70-2.05)	2.43(2.08-2.82)	598(3.82)	435(4.33)	163(2.92)
No	Ref.	Ref.	Ref.	Ref.	Ref.	Ref.	13 811(1.04)	8891(1.43)	4920(0.69)
**Cancer**									
Yes	1.27(1.00-1.58)	1.07(0.81-1.37)	1.85(1.01-3.05)	0.80(0.63-1.00)	0.80(0.61-1.02)	0.77(0.45-1.22)	63(1.35)	48(1.58)	15(0.92)
No	Ref.	Ref.	Ref.	Ref.	Ref.	Ref.	14 346(1.07)	9278(1.47)	5068(0.71)
**CVD**									
Yes	1.68(1.30-2.12)	1.59(1.08-2.24)	2.00(1.21-3.08)	1.24(0.96-1.58)	1.08(0.73-1.55)	1.34(0.95-1.84)	66(2.11)	24(2.29)	42(2.02)
No	Ref.	Ref.	Ref.	Ref.	Ref.	Ref.	14 343(1.07)	9302(1.47)	5041(0.71)
**Injury**									
Yes	2.52(1.86-3.32)	2.28(1.98-2.61)	2.60(2.32-2.91)	1.63(1.45-1.82)	1.50(1.30-1.72)	1.94(1.58-2.37)	261(2.70)	173(3.25)	88(2.02)
No	Ref.	Ref.	Ref.	Ref.	Ref.	Ref.	14 148(1.06)	9153(1.46)	4995(0.70)
**COVID-like**									
Yes	8.14(7.32-9.03)	6.62(5.86-7.46)	9.35(6.99-12.2)	4.82(4.32-5.37)	4.67(4.11-5.28)	5.31(4.22-6.59)	326(7.81)	246(8.69)	80(5.97)
No	Ref.	Ref.	Ref.	Ref.	Ref.	Ref.	14083(1.05)	9080(1.44)	5003(0.70)
**Other somatic**									
Yes	3.08(2.83-3.34)	2.45(2.22-2.69)	4.31(3.49-5.27)	1.83(1.68-1.99)	1.73(1.57-1.90)	2.14(1.80-2.53)	507(3.14)	375(3.43)	132(2.52)
No	Ref.	Ref.	Ref.	Ref.	Ref.	Ref.	13 902(1.04)	8951(1.44)	4951(0.70)

Table note: * including cases where information was missing, † married/cohabitant, ‡ from work and work-related activities

Supplementary Table 2: Unadjusted (crude) (columns 2-4) and adjusted (columns 5-7) odds ratios (OR) with 95% confidence interval (CI) for having had at least one incident SA-spell due to COVID-19 diagnosis in 2020 as outcome; including socio-demographic and work-related characteristics as well as SA-diagnoses in 2019 as factors; for all and stratified by sex. Frequencies (n) and proportion (%) (columns 8-10) of people in each category with the outcome of having had at least one incident SA-spell due to COVID-19 diagnosis in 2020, for all and stratified by sex. (Reported proportions are calculated based on all people in each category, i.e., for the total the denominator was n=1 347 778).

	**--------------------------- Crude ------------------------**			**------------------------- Adjusted --------------------- -**			**--------------- Frequencies ---------------**		
**Factors 2019**	**All OR(95 % CI)**	**Women OR(95 % CI)**	**Men OR(95 % CI)**	**All OR(95 % CI)**	**Women OR(95 % CI)**	**Men OR(95 % CI)**	**All n(%)**	**Women n(%)**	**Men n(%)**
**Total**									
**Sex**									
Woman	Ref	-	-	Ref	-	-	3841(0.61)	-	-
Man	0.52(0.50-0.55)	-	-	0.90(0.85-0.95)	-	-	2282(0.32)	-	-
**Age group (years)**									
19-24	0.50(0.41-0.61)	0.47(0.37-0.58)	0.44(0.29-0.65)	0.43(0.35-0.53)	0.46(0.35-0.58)	0.35(0.22-0.52)	106(0.20)	81(0.27)	25(0.11)
25-34	0.68(0.62-0.74)	0.63(0.57-0.70)	0.73(0.62-0.84)	0.63(0.58-0.70)	0.64(0.57-0.72)	0.60(0.51-0.71)	796(0.26)	533(0.35)	263(0.17)
35-44	Ref	Ref	Ref	Ref	Ref	Ref	1298(0.37)	868(0.54)	430(0.23)
45-54	1.58(1.48-1.70)	1.48(1.36-1.61)	1.81(1.61-2.03)	1.77(1.65-1.90)	1.61(1.47-1.76)	2.13(1.89-2.41)	2177(0.59)	1354(0.80)	823(0.41)
55-64	1.80(1.68-1.94)	1.58(1.44-1.73)	2.27(2.02-2.56)	1.98(1.82-2.16)	1.69(1.51-1.89)	2.53(2.20-2.91)	1662(0.70)	964(0.89)	698(0.54)
65-68	0.53(0.42-0.66)	0.40(0.29-0.54)	0.80(0.58-1.09)	0.85(0.67-1.06)	0.63(0.45-0.86)	1.23(0.87-1.69)	83(0.33)	41(0.36)	42(0.30)
**Level of education**									
Elementary (<10 years)*	1.07(0.94-1.23)	0.78(0.62-0.98)	1.60(1.35-1.90)	1.22(1.05-1.40)	0.97(0.76-1.22)	1.37(1.14-1.63)	220(0.44)	74(0.44)	146(0.43)
High school (10-12 years)	1.45(1.38-1.53)	1.41(1.32-1.50)	1.54(1.42-1.68)	1.26(1.19-1.33)	1.21(1.13-1.30)	1.34(1.22-1.47)	2849(0.57)	1789(0.75)	1060(0.40)
University (>12 years)	Ref	Ref	Ref	Ref	Ref	Ref	3053(0.38)	1978(0.52)	1075(0.26)
**Country of birth**									

Sweden	Ref	Ref	Ref	Ref	Ref	Ref	4372(0.38)	2687(0.5)	1685(0.27)
Other Nordic countries	1.56(1.32-1.83)	1.42(1.15-1.72)	1.62(1.21-2.13)	1.26(1.06-1.48)	1.22(0.99-1.49)	1.38(1.03-1.82)	151(0.63)	101(0.75)	50(0.47)
Other EU27 countries	1.33(1.16-1.52)	1.38(1.16-1.62)	1.20(0.94-1.51)	1.31(1.14-1.50)	1.31(1.10-1.55)	1.32(1.03-1.66)	217(0.53)	145(0.72)	72(0.34)
Rest of the world*	2.98(2.80-3.16)	2.93(2.72-3.16)	2.86(2.58-3.16)	2.24(2.10-2.39)	2.11(1.95-2.29)	2.55(2.28-2.84)	1383(1.15)	908(1.49)	475(0.79)
**Family situation**									
Partner†, no children at home	Ref	Ref	Ref	Ref	Ref	Ref	1527(0.57)	890(0.72)	637(0.45)
Partner†, children at home	0.74(0.69-0.79)	0.80(0.74-0.87)	0.67(0.61-0.74)	1.02(0.94-1.10)	1.09(0.99-1.21)	0.89(0.79-1.01)	2085(0.40)	1271(0.54)	814(0.28)
Single, no children at home*	0.75(0.71-0.81)	0.81(0.74-0.88)	0.68(0.61-0.75)	0.96(0.89-1.03)	1.04(0.95-1.14)	0.88(0.78-0.99)	2072(0.42)	1303(0.57)	769(0.29)
Single, children at home	1.27(1.14-1.42)	1.26(1.12-1.42)	0.74(0.56-0.96)	1.15(1.02-1.29)	1.23(1.08-1.41)	0.84(0.64-1.09)	438(0.69)	377(0.86)	61(0.31)
**Type of living area**									
Rural area	0.75(0.69-0.82)	0.73(0.66-0.81)	0.79(0.69-0.91)	0.76(0.72-0.80)	0.75(0.70-0.81)	0.76(0.69-0.83)	3446(0.51)	2196(0.68)	1250(0.35)
Town and suburb	0.80(0.76-0.85)	0.80(0.75-0.86)	0.83(0.76-0.91)	0.66(0.61-0.72)	0.65(0.58-0.73)	0.66(0.57-0.76)	2035(0.41)	1248(0.54)	787(0.29)
City	Ref	Ref	Ref	Ref	Ref	Ref	641(0.69)	397(0.50)	244(0.28)
**Company size**									
Micro (1-9)	Ref	Ref	Ref	Ref	Ref	Ref	493(0.33)	279(0.40)	214(0.27)
Small (10-49)	1.15(1.03-1.29)	1.20(1.04-1.39)	1.11(0.95-1.31)	1.18(1.05-1.31)	1.13(0.98-1.31)	1.23(1.04-1.45)	978(0.41)	550(0.48)	428(0.30)
Medium sized (50-249)	1.20(1.08-1.33)	1.26(1.10-1.45)	1.12(0.96-1.32)	1.18(1.06-1.32)	1.14(0.99-1.31)	1.25(1.06-1.47)	1180(0.40)	692(0.50)	488(0.31)
Large (≥250)	1.63(1.48-1.79)	1.90(1.68-2.15)	1.26(1.09-1.46)	1.46(1.32-1.61)	1.48(1.30-1.68)	1.39(1.19-1.61)	3432(0.54)	2293(0.75)	1139(0.34)
Missing info.	0.63(0.16-1.66)	1.07(0.26-2.80)	0.00(0.00-0.00)	0.61(0.15-1.59)	0.92(0.23-2.43)	0.00(0.00-0.00)	<10(0.27)	<10(0.42)	<10(0.00)
**Branch of industry**									
Services	Ref	Ref	Ref	Ref	Ref	Ref	1870(0.29)	952(0.33)	918(0.26)
Manufacturing	0.88(0.80-0.96)	0.78(0.67-0.90)	0.99(0.88-1.11)	0.84(0.77-0.93)	0.75(0.64-0.87)	0.87(0.77-0.97)	631(0.25)	205(0.26)	426(0.25)
Commerce and hospitality	1.35(1.21-1.49)	1.31(1.14-1.50)	1.32(1.13-1.55)	1.21(1.09-1.34)	1.24(1.08-1.42)	1.14(0.96-1.33)	443(0.39)	262(0.43)	181(0.34)
Transport	1.19(1.01-1.38)	1.06(0.82-1.35)	1.32(1.07-1.61)	1.05(0.89-1.22)	0.95(0.73-1.21)	1.07(0.86-1.31)	170(0.34)	67(0.35)	103(0.34)
Construction and installation	1.09(0.94-1.26)	0.85(0.63-1.12)	1.29(1.08-1.52)	1.13(0.97-1.30)	0.87(0.64-1.14)	1.20(1.00-1.42)	201(0.32)	49(0.28)	152(0.33)
Care and education	4.53(4.27-4.80)	4.35(4.03-4.69)	3.65(3.27-4.07)	2.94(2.76-3.14)	3.16(2.91-3.43)	2.41(2.14-2.71)	2770(1.29)	2276(1.42)	494(0.93)

Info. is missing	1.43(1.02-1.94)	1.67(1.14-2.36)	0.83(0.38-1.55)	1.42(0.78-2.35)	1.8(0.90-3.19)	0.84(0.21-2.19)	38(0.41)	>28(0.55)	<10(0.21)
**Income‡**									
0.18-<4 PBA	2.47(2.10-2.92)	2.00(1.57-2.58)	1.92(1.46-2.51)	2.19(1.84-2.61)	1.82(1.42-2.37)	2.34(1.75-3.10)	90(0.33)	337(0.52)	15(0.30)
4-<7.5 PBA	4.18(3.64-4.84)	3.54(2.83-4.49)	3.03(2.50-3.70)	3.05(2.63-3.55)	2.57(2.04-3.28)	3.32(2.70-4.10)	424(0.45)	1488(0.91)	87(0.47)
7.5-<10 PBA	3.01(2.61-3.47)	2.51(2.00-3.18)	2.81(2.35-3.40)	2.65(2.29-3.08)	2.13(1.70-2.72)	3.22(2.67-3.92)	1891(0.76)	1171(0.65)	403(0.44)
10-<12.5 PBA	2.02(1.74-2.34)	1.74(1.38-2.23)	2.04(1.69-2.47)	2.01(1.73-2.34)	1.61(1.27-2.07)	2.30(1.91-2.80)	1897(0.55)	469(0.45)	726(0.32)
12.5-<15 PBA	1.48(1.26-1.75)	1.32(1.01-1.75)	1.53(1.25-1.89)	1.53(1.30-1.81)	1.31(1.01-1.73)	1.64(1.34-2.03)	1014(0.37)	165(0.34)	545(0.24)
15-<17.5 PBA	1.15(0.94-1.41)	1.03(0.73-1.44)	1.21(0.93-1.56)	1.19(0.97-1.45)	1.04(0.74-1.46)	1.26(0.98-1.63)	431(0.27)	59(0.27)	266(0.19)
≥17.5 PBA	Ref	Ref	Ref	Ref	Ref	Ref	211(0.18)	77(0.26)	134(0.16)
**SA diagnosis 2019**									
**Depression**									
Yes	2.07(1.63-2.58)	1.75(1.32-2.26)	2.24(1.39-3.39)	1.59(1.25-1.99)	1.5(1.13-1.95)	1.77(1.09-2.68)	75(0.93)	55(1.05)	20(0.71)
No	Ref	Ref	Ref	Ref	Ref	Ref	6048(0.45)	3786(0.60)	2262(0.32)
**Anxiety**									
Yes	2.17(1.63-2.82)	1.71(1.21-2.33)	2.73(1.57-4.38)	1.73(1.29-2.26)	1.53(1.07-2.10)	2.28(1.30-3.67)	52(0.98)	37(1.03)	15(0.86)
No	Ref	Ref	Ref	Ref	Ref	Ref	6071(0.45)	3804(0.60)	2267(0.32)
**Exhaustion**									
Yes	1.71(1.37-2.10)	1.52(1.20-1.90)	1.32(0.74-2.14)	1.41(1.13-1.74)	1.48(1.16-1.86)	1.08(0.61-1.77)	88(0.77)	74(0.91)	14(0.42)
No	Ref	Ref	Ref	Ref	Ref	Ref	6035(0.45)	3767(0.60)	2268(0.32)
**Other stress-related**									
Yes	2.26(1.89-2.68)	1.99(1.63-2.39)	1.73(1.06-2.63)	1.66(1.38-1.97)	1.72(1.41-2.07)	1.31(0.80-2.01)	129(1.01)	110(1.18)	19(0.55)
No	Ref	Ref	Ref	Ref	Ref	Ref	5994(0.45)	3731(0.60)	2263(0.32)
**Other mental**									
Yes	2.08(1.30-3.15)	2.24(1.37-3.41)	0.44(0.03-1.94)	1.63(1.00-2.49)	1.99(1.20-3.09)	0.35(0.02-1.54)	20(0.94)	>9(1.34)	<10(0.14)
No	Ref	Ref	Ref	Ref	Ref	Ref	6103(0.45)	3822(0.60)	2281(0.32)
**Musculoskeletal**									

Yes	3.05(2.65-3.50)	2.49(2.10-2.93)	3.63(2.79-4.63)	1.64(1.42-1.89)	1.48(1.24-1.75)	2.17(1.66-2.78)	210(1.34)	147(1.46)	63(1.13)
No	Ref	Ref	Ref	Ref	Ref	Ref	5913(0.44)	3694(0.59)	2219(0.31)
**Cancer**									
Yes	1.28(0.85-1.82)	1.20(0.76-1.77)	0.96(0.34-2.08)	0.83(0.55-1.20)	0.95(0.60-1.41)	0.50(0.16-1.18)	27(0.58)	>17(0.72)	<10(0.31)
No	Ref	Ref	Ref	Ref	Ref	Ref	6096(0.45)	3819(0.61)	2277(0.32)
**CVD**									
Yes	1.55(0.99-2.30)	1.90(1.01-3.20)	1.51(0.75-2.66)	1.20(0.76-1.78)	1.47(0.78-2.49)	0.91(0.46-1.61)	22(0.70)	12(1.14)	10(0.48)
No		Ref					6101(0.45)	3829(0.61)	2272(0.32)
**Injury**									
Yes	2.24(1.82-2.72)	2.20(1.72-2.77)	1.96(1.31-2.81)	1.45(1.17-1.77)	1.5(1.17-1.89)	1.35(0.88-1.96)	97(1.00)	70(1.31)	27(0.62)
No	Ref	Ref	Ref	Ref	Ref	Ref	6026(0.45)	3771(0.60)	2255(0.32)
**COVID-like**									
Yes	3.94(3.09-4.93)	3.46(2.64-4.45)	3.55(2.03-5.69)	2.41(1.88-3.02)	2.45(1.86-3.16)	2.23(1.28-3.60)	73(1.75)	58(2.05)	15(1.12)
No	Ref	Ref	Ref	Ref	Ref	Ref	6050(0.45)	3783(0.60)	2267(0.32)
**Other somatic**									
Yes	2.77(2.40-3.19)	2.45(2.08-2.87)	2.48(1.79-3.34)	1.76(1.52-2.03)	1.79(1.51-2.10)	1.60(1.15-2.15)	198(1.22)	157(1.44)	41(0.78)
No	Ref	Ref	Ref	Ref	Ref	Ref	5925(0.44)	3684(0.59)	2241(0.32)

Table note: * including cases where information was missing, † married/cohabitant, ‡ from work and work-related activities
